# Supplementary figures and images for: Cluster detection with random neighbourhood covering: Application to invasive Group A Streptococcal disease
Source: PLoS Comput Biol. 2022 Nov 30;18(11):e1010726. doi: 10.1371/journal.pcbi.1010726 (PMC9744322; doi:10.1371/journal.pcbi.1010726)

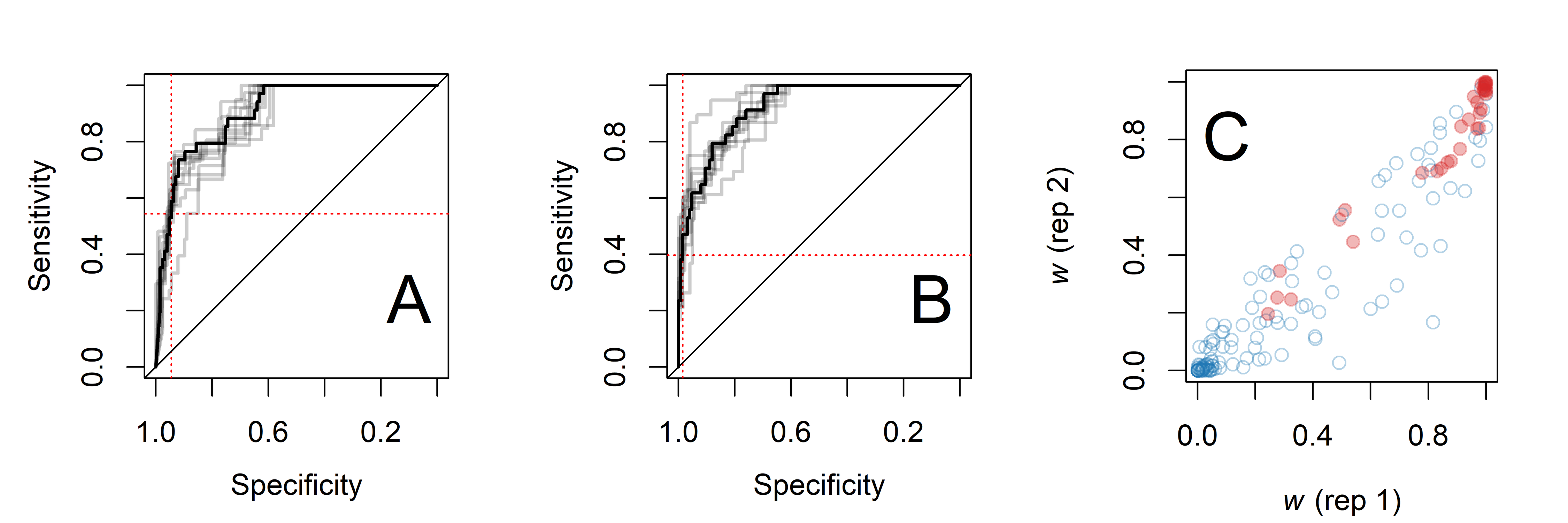

Supplement: S1 Fig — A-B ROC curves (solid black line) of RaNCover predictions over two replicates, obtained using different cylinder volumes. Solid grey curves are from ROCs obtained by means of bootstrapping. The dotted red lines intercept the values of sensitivity (horizontal lines, sensitivities equal to 0.54 and 0.40 for A and B, respectively) and specificity (vertical lines, specificity equal to 0.94 and 0.98 for A and B, respectively) obtained from setting 1−α = 0.95 as a discrimination threshold. C Correlation plot between warning scores from the two replicates. Red markers correspond to true epidemic simulation events. (TIF) [file pcbi.1010726.s001.tif]

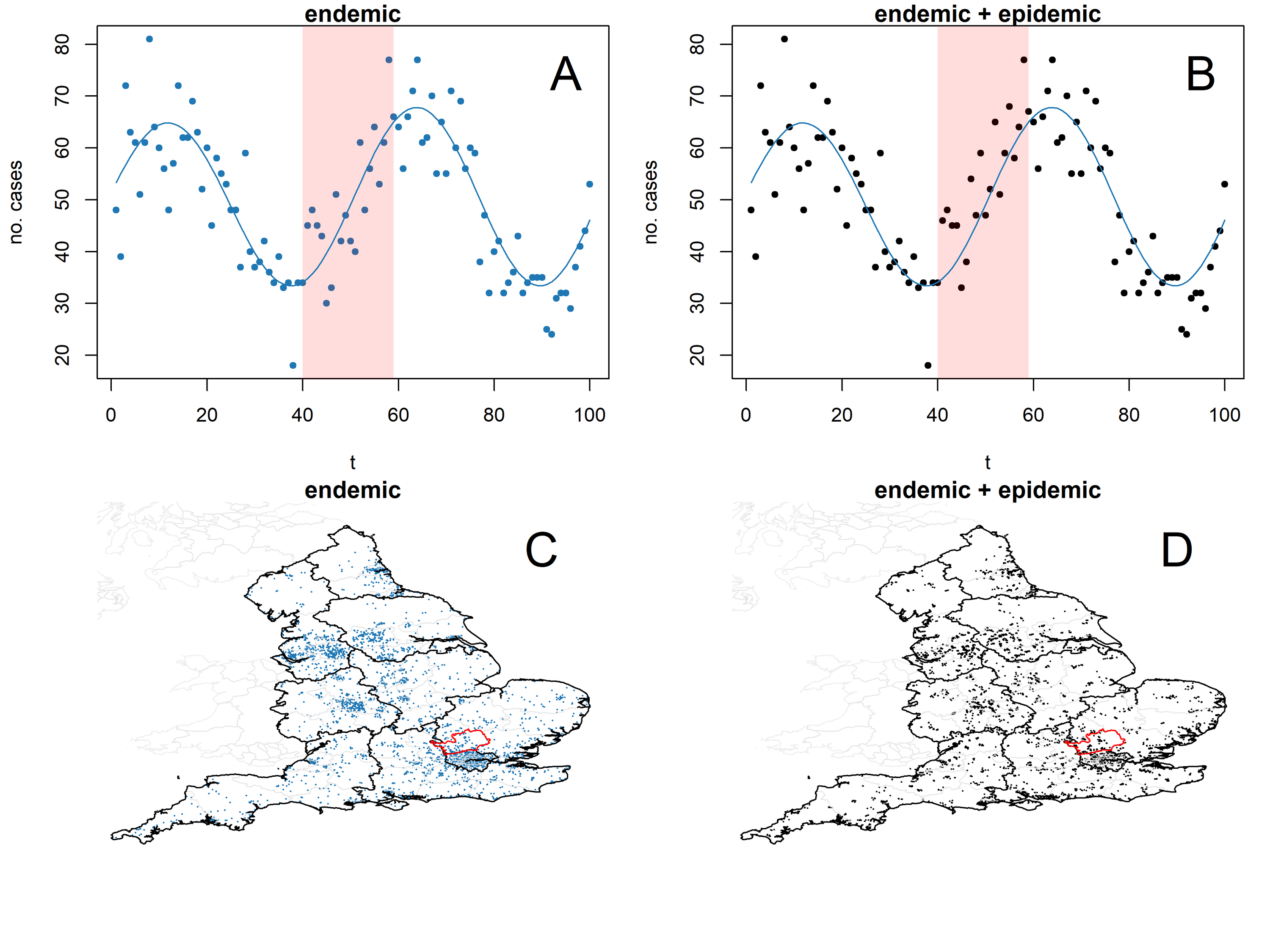

Supplement: S2 Fig — Illustration of simulation-experiment data aggregated and projected to time (A-B) and geography (C-D). Cases are simulated from a Poisson point process with endemic intensity function fitted on true iGAS data, thus encoding for seasonality (A) and population density (C). Other cases representing a small outbreak occurring from t = 40 to t = 59 in St Albans (Hertfordshire, highlighted areas in A,B,C, and D) are included in the synthetic dataset; the overall temporal and geographical patterns are not affected (B and D), yet the algorithm is able to spot the anomaly (Fig 6, main text). Maps created with Sf [53] using shapefiles from the GADM database (www.gadm.org) and the Ordnance Survey Data Hub (osdatahub.os.uk). Same data embedded in a two-dimensional plane using t-SNE are illustrated in S4 Fig. (TIF) [file pcbi.1010726.s002.tif]

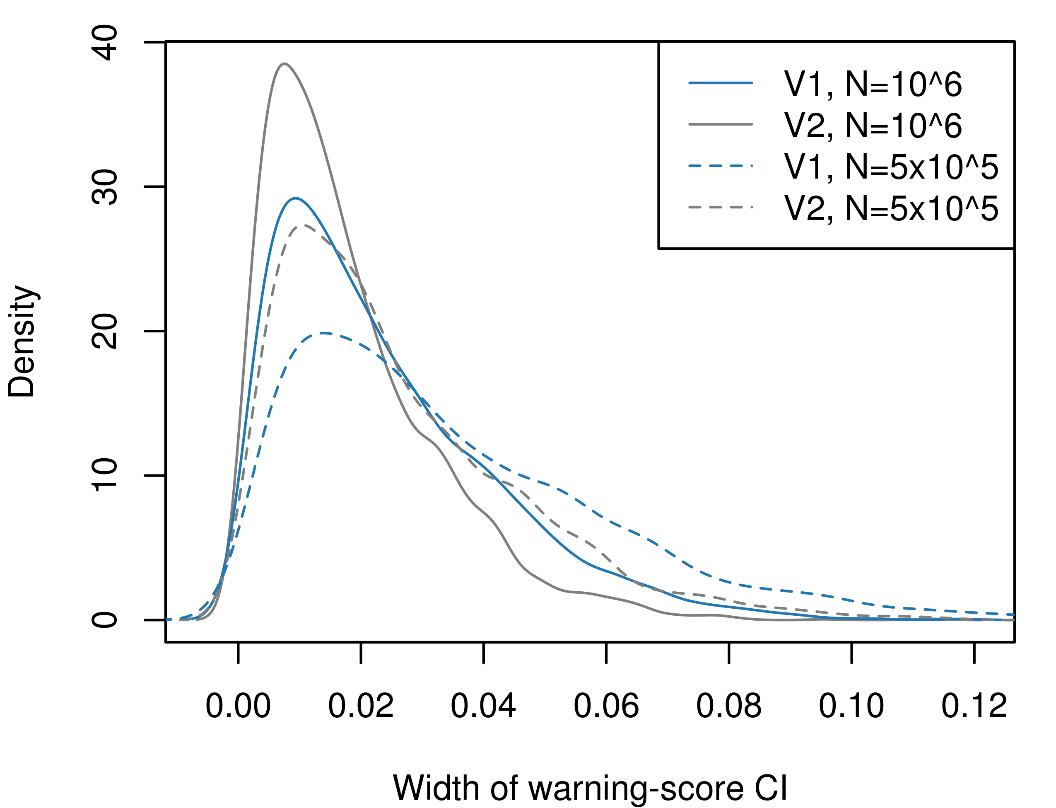

Supplement: S3 Fig — Widths of confidence intervals (CIs) of warning scores in simulation experiment for two choices of total number of random cylinders drew (N = 106 and N = 5×10^5^, solid and dashed lines, respectively) and two choices of cylinders’ volume (V1 and V2 = V1×1.4, blue and grey lines, respectively). The warning scores are proportions of flagged cylinders and appropriate CIs are Wilson’s for binomial proportions. Increasing N or increasing the volumes, the numbers at numerator and denominator of the proportion increase and the confidence in its estimate improves. (TIF) [file pcbi.1010726.s003.tif]

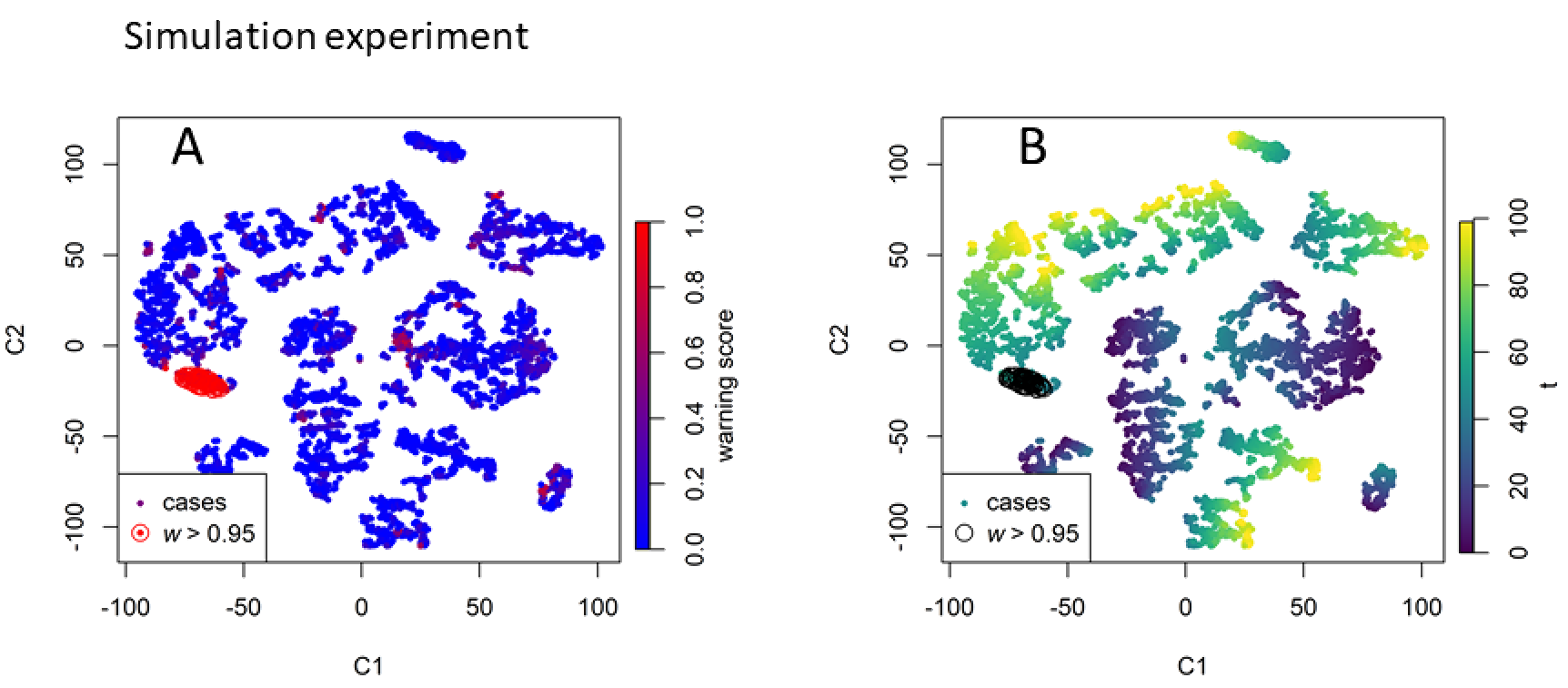

Supplement: S4 Fig — Each simulated record is identified by a point of coordinates C1 and C2. The points are coloured by their warning scores ((A) 0 to 1, blue to red, highlighting the presence of a bright red cluster of points with high warning scores w>0.95) and by their record time (B), showing that t-SNE also preserves temporal proximity. (TIF) [file pcbi.1010726.s004.tif]
