# Supplementary material for: Cluster detection with random neighbourhood covering: Application to invasive Group A Streptococcal disease
Source: PLoS Comput Biol. 2022 Nov 30;18(11):e1010726. doi: 10.1371/journal.pcbi.1010726 (PMC9744322; doi:10.1371/journal.pcbi.1010726)
Supplement: Attachment — Submitted filename: response to reviewers.pdf [file pcbi.1010726.s005.pdf]

Dear Editor,

We would like to thank the three reviewers for carefully studying our manuscript and for providing constructive comments for improvements and clarity. We have significantly revised our manuscript based on their input as described in the point-by-point response below. In the revised manuscript we also included two new supplemental figures and cited 5 new references (Refs. [39,40,52,53,54] in the revised manuscript).

Reviewer #1:

The authors introduce a new method (RaNCover) intended, after further validation, to be applied for spatiotemporal surveillance of communicable diseases. I review from the perspective of a public health practitioner and epidemiologist with experience applying the scan statistics in SaTScan for prospective outbreak detection. I advise this paper also be evaluated by a reviewer with more biostatistical expertise than I.

1. P. 7: "The general problem we address here is to assess whether a single recorded infection might be part of a cluster or is consistent with a baseline of sporadic cases:" this seems like a non sequitur. Could the authors please clarify the logical flow from the rest of the Introduction to the statement of the practical problem this new method was created to address? In jurisdictions where disease counts are so sparse that RaNCover should be run after every new case is reported, then there can be eyes on every case to assess whether an outbreak is unfolding. It seems that running prospective analyses with data aggregated to a daily time interval should be sufficient for timely iGAS outbreak detection.

We thank the reviewer for these useful and practical comments. In the revised manuscript Introduction *(page 3, line 83)*, the full sentence in its context reads: "*To help identify clusters of iGAS which share the same emm type and are epidemiologically linked by geography and in time, we developed the random neighbourhood covering (RaNCover) approach. As an effective and automated algorithm, it would be complementary to what local health protection or public health teams can offer in the initial stages of outbreak detection, and may be of particular use with outbreaks which cross regional or team borders that might otherwise be difficult to detect. The general problem RaNCover addresses is to classify a single recorded infection as being part of a cluster or being consistent with a baseline of sporadic cases".* This makes more clear what are the topical and the general problems this method was created to address. Since the RaNCover approach can be applied prospectively as well as retrospectively and this difference is not key to the approach itself, we opted not to include these notions in the "Introduction" section.

2. P. 9: "standard false discovery rate = 0.05" This is standard for retrospective analyses, but for repeated, prospective analyses as the authors propose, this will cause too much false signaling. Recurrence intervals > 1 year are more standard thresholds. (See, e.g., SaTScan user manual, "recurrence interval.")

We feel that the reviewer might not have fully appreciated the subtleties of our method; we have now re-worded the "Methods" section (page 5, lines 172-190) to make this more clear.

We agree with the reviewer, that if we just used a single cylinder to test the number of cases in a time-space volume, then if cases are random and unclustered (our null model) the number of cases in each cylinder is a Poisson sample. Hence our false positive rate *alpha* would give an error every 5% of samples - so with one test per day we would expect 20 such false signals in a year. We can think of this as a warning flag for each cylinder.

However, our method uses multiple cylinders to generate a warning flag for a cluster. We are interested in the proportion of cylinders that have individual warning flags; we only set a warning for a geographic cluster if at least 95% of the cylinders covering a case have individual warning flags -- that is if 95% are above the 0.95-quantile value. In the null model we expect just 5% of cylinders to have a warning, and for this to be independent for each cylinder, so even with just 20 cylinders, the probability of generating a false flag is astronomically small (probability of 19 out of 20 cylinders having a flag is 3.6×10^{-24}).

Therefore by considering the state of multiple cylinders, we have a method that is extremely robust to random fluctuations and is highly unlikely to identify a geographic cluster from random case data.

3. P. 15, "Prospective analysis and emm typing delay" section: Do the authors propose health departments implement one prospective analysis for all iGAS data regardless of typing, a separate prospective analysis for every emm type, and/or a separate prospective analysis for every emm type and all untyped cases? For operational simplicity, is it possible to run one daily analysis for iGAS, accounting for both emm type (if available) and untyped cases?

Yes, it is indeed possible to run one daily analysis and analyse all emm types and the untyped cases in parallel, please see also reply to the comment 11 of the same reviewer.

4. Could the authors please clarify if and how iGAS seasonality patterns vary across emm types?

We assumed that the baselines of all emm types have the same seasonal pattern (more cases expected during the winter than the summer). To the best of our knowledge (the UK-HSA topic lead is an author on this paper), there is no evidence that seasonality differs by emm type.

5. Could the authors provide recommendations for choosing the length of baseline period?

Its choice depends on the temporal pattern of the disease we are interested in. Since it is known that iGAS shows seasonal patterns, we suggest that this length ideally needs to be long enough to capture seasonality. This has now been explained in the updated manuscript (page 7, line 272). We argue that the longer the length of this period, the better the estimate (unless during this time there are changes in the way cases are reported). We estimated the baseline for iGAS using all available data from the beginning of the study.

6. Is it correct that iGAS disease data were obtained for 2015-2020, but the population denominators were from 2011 (P. 11)? If so, why did the authors use outdated estimated population sizes?

Yes, that is correct. Using population densities from the 2011 census is a convenience choice. To the best of our knowledge, these are the most-recent publicly available census data on population density in England at the required resolution (postcode level). RaNCover is not limited to such a choice, please also see our response to the next question.

7. Could the authors please comment on strengths and limitations for using RaNCover with population denominator data (which can quickly become outdated for small areas), when there are alternative methods readily available (e.g., space-time permutation scan statistic, Ref #19) that use disease counts in the baseline period to establish expected case counts, with no requirement for population denominators?

We think that methods for outbreak detection should take spatial population density data into account or otherwise suffer from heavy biases towards locations with high population density, such as metropolitan areas, although we are aware that this may not work with any surveillance data.

Key question is: how to estimate such a spatial component when population-at-risk data is not available or not appropriate? The authors of reference [Kulldorff2005] (Ref #19 above) condition on the observed marginals to obtain the expected number of cases (to quote [Kulldorff2005] "*Since we do not have population-at-risk data, the expected must be calculated using only the cases.*", please see also equation (2) in the same reference). We could have done the same here instead of estimating the spatial component of the baseline from census data. However, given the overall sparsity of iGAS cases, we believe that using census data provides better estimates than conditioning on the observed marginals and we do not see our choice as a weakness for iGAS.

To make this point clear we included the following text (page 5, line 155): "*We make the simplifying assumption that [the spatial factor] is proportional to the population density, reflecting the fact that the number of observed episodes in an area is likely to increase with its population size – although [the spatial factor] could be matched to historical infection data if these were plentiful. This population-based weighting is arguably the most important factor in the spatial distribution of the episodes, although, across geographical areas, other factors such as the climatic zone or disparities in access to health care could affect the baseline*".

Further, in the *Discussion* section (page 14, line 496) we added "*For this disease, we estimated the baseline taking into account population denominator data and iGAS seasonality* " to highlight that we used population denominator data in this specific case study, while RaNCover is more general.

8. In the statements on ethics and software and reproducibility, I suggest the authors clarify the study also used synthetic data, as posted to their GitHub site.

We edited the "Software and reproducibility" section accordingly: "*Analyses were carried out using R ([42], version 3.4.3). [...]. All software (also including codes for generation of synthetic data) is archived online at https://github.com/mcavallaro/outbreak-detection.*"

9. Fig. 5: could the authors please comment re: whether the large spike in 2018 above baseline was a true outbreak? What is the explanation for this increase?

We updated the caption of Fig. 5 accordingly. 2018 was a year of heightened transmission across England rather than a single outbreak, as reported in https://webarchive.nationalarchives.gov.uk/ukgwa/20220202090104/https:/www.gov.uk/government/publications/group-a-streptococcal-infections-activity-during-the-2017-to-2018-season

10. P. 13: re: the SaTScan analysis "not accounting for seasonality, which would further increase the computational burden:" Please clarify why the authors chose to apply the discrete Poisson model. The space-time permutation model (Ref #19) would have accounted for purely spatial and purely temporal clusters such as seasonal effects.

A Poisson point process models infection cases from a population at risk and represents well our case study. This is also stated in the SaTScan(™) software manual (although we are aware that the scan statistics and SaTScan(™) software allows different designs). Since our RaNCover method is based on the Poisson model, to make a valid comparison between SaTScan(™) and RaNCover, we performed cluster detection with SaTScan(™) using the discrete Poisson model. This clarifies our choice.

Also, it appears to us that SaTScan with discrete Poisson model can in principle account for seasonality but with substantially increased computational burden. We believe this computational burden is intrinsic to SaTScan(™) and we expect that SaTScan(™) with space-time permutation model would suffer from the same issue.

11**. P. 16, "Compared to existing methods, [RaNCover] excels in execution speed and flexibility." First, 3 hours is not a prohibitively long run-time, since programs can be automated and run overnight to be complete by open of business. Second, could the authors please clarify how RaNCover is more flexible than the scan statistics, and how this flexibility adds practical value?**

We obtained those detailed RaNCover results in about 5 minutes instead of hours. This is a staggering increase in performance, which allows users and practitioners to scale up their analyses to more datasets or different emm types in parallel, and test under different conditions.

Including seasonality in the same discrete Poisson model in SaTScan(™) was prohibitively slow, at least with our hardware.

Increased flexibility also comes from the fact that RaNCover assesses single cases individually and thus can find clusters of cases distributed in an arbitrary shape (methods based on the scan statistics outputs an outbreak area of a determined shape such as a cylinder).

12. P. 17: "traditional surveillance methods typically require aggregating data over time and space:" In our applications of scan statistics, we aggregate to daily temporal resolution and (depending on the application) either census tract spatial resolution or the exact X-Y coordinates of the patient address (i.e., no spatial aggregation necessary). Please edit to avoid suggesting that other methods require use of "coarse-grained time windows or geographical regions." Further, it appears the authors aggregated to "patient postcode location" (P. 11), so it appears that unlike

some of the scan statistics, RaNCover has the limitation of requiring aggregating to geographical regions with accurate population denominator data.

To avoid suggesting that all other methods require use of "**coarse-grained time windows or geographical regions"** we included the following sentence in the discussion section (page 14, line 530) "*Similarly to SaTScan(™), our method can be used at arbitrary levels of geographical and temporal aggregation. RaNCover further provides a continuous warning score for each detected case, rather than for coarse-grained time windows or geographical regions, and yet remains able to highlight clusters of diseases.*" This is also to explain that RaNCover gives information on each single case, while SaTScan*(™)* output is an area where the outbreak is most likely to have occurred.

It is incorrect to state that RaNCover has the limitation of requiring aggregating to geographical regions with accurate population denominator data. This follows from its mathematical formulation and also was demonstrated in the first simulation experiment, used to detail and demonstrate our methodology in action into the "Methods" section.

13. Could the authors please explain more explicitly why the method is named "RaNCover," i.e., in what sense are neighborhoods being covered randomly?

The name of the method follows from notions in mathematics, in particular topology. More explicitly, by definition, a cover of a set A is a collection of sets whose union includes A as a subset. In the updated manuscript, we included the following explanation in the Introduction section (page 3, line 90): "*[...] we consider a neighbourhood of the point location and time of the recorded case and appropriately draw a random collection of spatio-temporal cylinders to cover it. Based on the properties of this collection of covering cylinders it is possible to perform classification.*"

Reviewer #2:

The study entitled "Cluster detection with random neighbourhood covering: application to invasive Group A Streptococcal disease" by Cavallaro et al. presents a very interesting approach to detect epidemic events based on spatio-temporal data. It demonstrates how the method can be applied to detect outbreaks of invasive GAS, integrating emm-typing information. The latter point is of special importance, as such level of detail has been previously ignored in several epidemiological models. Approaching the complexity of the real picture as much as possible, including delay of typing, is a merit of this study. The manuscript is clear and well written, and code is available.

We thank the reviewer for their positive comments.

Additional comments:

- Although the iGAS cases across England are confidential data, it is very helpful to be able to run the code of simulation_experiment_England.Rmd.

Unfortunately, the file including geographical location data (shape_file_new/Areas.shp) is not available… A comment implies that sharing of the UK boundaries shape files might not be allowed… Is it not?

In fact, we might not be allowed to redistribute all the .shp location data files. We uploaded a new script `simulation_experiment_England.Rmd` which runs the simulation experiment without these .shp files.

- The dimensionality reduction method 't-distributed stochastic neighborhood embedding' is used in most figures of the paper. Please give more in details regarding the algorithm used, the parameters considered (is it only the three variables x, y, time, or other variables?).

In the first paragraph of the section "Application to iGAS disease: Cluster analysis and data-point embedding" of the revised manuscript we explicitly stated that x, y, and time of detection are the variables considered (page 10, line 381). Further, to include more details, we stated (page 10, line 378) "*In other words, t-SNE is a general statistical-learning method for dimensionality reduction which here is used to yield a two-dimensional point for each point in the original three-dimensional space, in such a way that nearby events in space and time appear next to each other in the two-dimensional map.*".

- Linking the two previous points, would it be possible to add two panels to Fig. 6 displaying the simulation data as they are displayed in the A-B panels of the following figures? (it would be slightly redundant with the current panels B and C, so doing so in an additional supplementary figure might be the best)

In the revised manuscript, we included a new supplemental figure (Fig S3) with the data from the simulation experiment displayed as in the A-B panels of Figs 7-10.

- Equation 3) there is a parameter E, that is not mentioned in the following definition

We thank the referee for spotting this. We correctly reported the parameter values in the revised manuscript.

- Please define timeliness

We included a definition of timeliness in the *Methods* section (page 6, line 240) "*This measures the time elapsed between the case detection day and the day at which its warning score stabilises around its final value (corresponding to the retrospectively computed warning score).*".

- For four rare emm-types, outbreak clusters were identified, while for the only common emm-type examined, no outbreak cluster was identified. Is this a coincidence? Is there a specific reason why the other two common emm-type (emm-1.0 and emm-89.0) were not examined? Can the authors elaborate on detection efficiency depending on lambdam? and/or on the implication of an increasing lambdam in the years considered?

We did not examine emm-1.0 and emm-89.0 for brevity. Future work to incorporate the algorithm in practice will cover these and other common emm types. It is coincidental that

the common emm-type examined did not display outbreak custers and we do not expect that varying lambda_m alters the detection efficiency.

- "As anticipated in synthetic data..." the authors probably refer to figure 3 and 6E, and similarly in the legend of Fig S2, "the algorithm is able to spot the anomaly …" the authors probably refer to Fig 6

We really thank the referee for finding these typos. In the revised manuscript, we updated the labels accordingly.

- Fig. 12 the x-axes range and labels change in each panel make the figure difficult to read. Having same range/labels in B/D and highlight which portion of A/C they correspond to may facilitate understanding.

We edited Fig 12 B, and D accordingly and highlighted which portion of A they correspond to.

- Fig S2 makes an important point very clear, but adding a visual guide pointing towards the actual differences (e.g shading of the timeframe 40-60 hours and the region of St. Albans) would be helpful.

We edited Fig S2 accordingly.

Reviewer #3:

The topic addressed in this article is extremely important for epidemiological surveillance in general and for the detection of infectious disease clusters in particular. The introduction is very clear but the article suffers from three major shortcomings concerning the description of the method, the simulation study and the scripts.

The method would need to be structured in sub-sections and probably lacks a part to help users make choices on tuning parameters . For example, how to choose a value for N in practice?

The *Methods* section has now been reorganised in four sub-sections. To explain the impact of N, at page 6, line 186, of the updated manuscript, we also included: "[...] *This suggests raising a warning for the event **x** if the fraction of flagged subsets is greater than than a chosen threshold, where the statistical significance is tested by means of a binomial test for proportions. Wilson intervals [39] are used as 2.5\%-97.5\% confidence intervals (CIs) for the warning scores. As N increases, these CIs tighten and the test significancy improves, thus allowing practitioners to tune the parameter N and reach their desired significance level.*"

What does "on average greater than one" or "contains on average more than one basic event" mean? The choice of the volume constant is not clear.

We thank the reviewer for spotting this imprecision in the text, which we removed. Baseline events are endemic events. Its expected number in a volume is given by the integral of the baseline intensity (lambda) over the volume (see equation (2)). As a simplification, we consider instead the average intensity C over space and time, multiply it by the volume of

the cylinder V=pi*h*rho^2, and require that all cylinders have identical constant C*V, which approximates the number of baseline events in the generic cylinders. We set the value of C*V heuristically, since we demonstrated that its choice is not crucial for the results. At page 6, line 197, we edited the manuscript to clarify and explain how we have chosen the volume constant : "*The algorithm appears to be robust to the choice of this volume constant (see simulation experiments); its value is heuristically set to avoid excessively small volumes, expected to contain only one event, and excessively large volumes, which would contain more cases but might not represent well local information.*" The values of the cylinder constant used are reported at page 6, line 222, and page 9, line 330, for the demonstration and the full simulation experiment, respectively.

What is the impact of the choice of tau on the performance of the method?

Data collected during an initial time interval is used to estimate the null-model baseline for iGAS. We referred to this time interval as tau. The choice of tau depends on the temporal pattern of the disease we are interested in. For example, since it is known that iGAS shows seasonal patterns, we suggest that tau needs to be at least *long enough to capture seasonality, with longer tau arguably producing better estimates*. This has been explained in the updated manuscript (page 7, line 272).

The choice of tau regards the estimation of the baseline and does not impact the performance of the random neighbourhood cover method itself.

The simulation study is not well designed. The proposed method needs to be evaluated by a thorough simulation study, which could be described in detail in an appendix. In this respect, the authors can follow the recommendations proposed in this article: Using simulation studies to evaluate statistical methods (Morris, White, Crowther) Statistics in Medicine 2017 . A thorough simulation study is the only way to scientifically validate the proposed method and this is not the case in the manuscript.

We identified two aspects to improve in the simulation experiments and updated the revised manuscript accordingly. In the new version of the manuscript:

1) we have improved the reporting of the main simulation study. This study is now also accessible by means of the markdown document `simulation_experiment_England.Rmd` file (please see also reply to the next comment). Instead of including an appendix, we have expanded and clarified the "Methods" and the "Simulation study" sections with all important details.

2) we have included confidence intervals on the estimated warning scores (please see also our replies to the first comment of the same reviewer and to the second comment of the first reviewer). We also included an additional reference that details the simulation of spatial Poisson processes used for the generation of synthetic data [Baddeley2016]. We can confirm that our modelling methodology conforms with the approach detailed in Morris, White & Crowther.

The scripts that are available on Github do not allow to run the method. Some scripts (source programs) are missing and some data are also missing. It is thus not possible to test its behaviour using other real data. It would be very helpful if the authors would provide a main program that works with simulated data.

We have now uploaded on our GitHub repository working versions of `simulation_experiment_England.Rmd` (also available as an R script (main program) `simulation_experiment_England.R`), `simulation_experiment_ROCAUC.Rmd`, and `simulation_experiment_timeliness.Rmd`, which run the simulation experiments without geographical map files, which we might not be allowed to redistribute (please see also first comment from the second reviewer).

References

[Kleinman2004] Kleinman K, Lazarus R, Platt R. A generalized linear mixed models approach for detecting incident clusters of disease in small areas, with an application to biological terrorism. American Journal of Epidemiology, 159:217-24, 2004.3

[Kulldorff2005] Kulldorff M, Heffernan R,Hartman J, Assuncao R, Mostashari F (2005) A space–time permutation scan statistic for disease outbreak detection. PLoS Med 2(3): e59.

[Baddeley2016] Baddeley, A., E. Rubak, and R. Turner, *Spatial Point Patterns: Methodology and Applications with R.* Spatial Point Patterns: Methodology and Applications with R, 2016
