# Supplementary material for: Cluster detection with random neighbourhood covering: Application to invasive Group A Streptococcal disease
Source: PLoS Comput Biol. 2022 Nov 30;18(11):e1010726. doi: 10.1371/journal.pcbi.1010726 (PMC9744322; doi:10.1371/journal.pcbi.1010726)
Supplement: Attachment — Submitted filename: response_to_reviewers.docx [file pcbi.1010726.s006.docx]

Dear Editor,

We thank the reviewers for providing further constructive comments for improving and clarifying our manuscript. We have updated our manuscript based on their input and included two additional references ([18,19] in the main text), as also described in the point-by-point response below.

**Reviewer #1:**

**C1: I thank the authors for carefully considering my first round of comments. I reiterate that I review from the perspective of an applied public health practitioner and am not a statistician so I am perhaps not your primary audience.**

R1: We appreciate the reviewer’s interest in the topic and we thank them for their comments.

**C2: I would like to comment on the responses to Reviewer #1 based on my experience with SaTScan because the contrasts with SaTScan in this manuscript can support public health practitioners in choosing which methods to apply in practice:**

**#7, re: “We think that methods for outbreak detection should take spatial population density data into account or otherwise suffer from heavy biases towards locations with high population density, such as metropolitan areas, although we are aware that this may not work with any surveillance data.”**

**- In the absence of large population movement during the study period, areas that have large case counts during the temporal window of interest because of high population density also have large case counts in the baseline period, so the expected number of cases for any area implicitly accounts for the population distribution, avoiding the “heavy biases” the authors mention. This has been my experience in applying the space-time permutation scan statistic.**

R2: We agree with the reviewer that the bias discussed above is not necessarily “heavy”; in fact, with enough baseline data it is typically possible to see that high population density also have large case counts, and therefore it is not always necessary to include population denominator. In fact, in our manuscript (line 157-161 of revised manuscript) when we derived the baseline for iGAS, we stated “*We make the simplifying assumption that 𝜆_0_(𝑥, 𝑦) is proportional to the population density, reflecting the fact that the number of observed episodes in an area is likely to increase with its population size –* ***although 𝜆_0_(𝑥, 𝑦) could be matched to historical infection data if these were plentiful.***” For iGAS, there is not enough data to estimate population density and we therefore used census population data.

We arguably should have avoided to use the word “heavy” in the text quoted by the reviewer; that sentence is taken from our previous point-to-point response and the manuscript remains correct in this respect (see highlighted sentence above). We also stress that this aspect only regards the way the baseline is computed. While for iGAS we believe that using population data is useful, RaNCover can use a baseline estimated differently. As an example, when we illustrated by means of synthetic data and baseline (line 211 and Fig 2) we used a given baseline (population denominator was not necessary).

**C3: - This relates to my main remaining question after reading through the revision: could the authors please provide more intuition about how RaNCover is able to classify every single recorded infection as cluster-related vs. sporadic for areas with high population density where the baseline level of endemic disease is high?**

R3: Broadly speaking, a classification method outputs a value between 0 and 1 that corresponds to the probability of belonging to a given class (e.g., 0.6 corresponds to 60% chance of being cluster-related). This is what we achieve with RaNCover, which outputs a warning score w(**x**) for each observed case **x**. In area with high population density where the baseline level of endemic disease is high, there will be more cases to classify but the underlying intuition remains the same: to obtain w(**x**), RaNCover averages the information from all the cylinders that contains the spatio-temporal coordinate **x**. In our minor revision, we clarified this into the discussion section (line 481).

“[…] *drawing and inspecting many cylinders covering each event* ***x*** *permits defining a continuous warning score w* ***between 0 and 1*** *specific to* ***x****.* ***This permits classification of cases in two groups, one for outbreak and one for baseline, with w corresponding to the probability that a case is outbreak-related.***”

When the cases are densely distributed, it happens that these cylinders overlap and their information is shared, thus yielding correlated warning scores, as also noted in the manuscript (line 203 of revised manuscript).

**C4: #10, re: “Also, it appears to us that SaTScan with discrete Poisson model can in principle account for seasonality but with substantially increased computational burden. We believe this computational burden is intrinsic to SaTScan and we expect that SaTScan with space-time permutation model would suffer from the same issue.”**

**- Per the SaTScan user manual: “The space-time permutation model automatically adjusts for purely spatial and purely temporal clusters [seasonal trends are purely temporal clusters]. For the discrete Poisson model, purely temporal and purely spatial clusters can be adjusted for in a number of different ways [options for temporal trend adjustments, e.g., for seasonality, include nonparametric, log-linear, or log-quadratic].” I believe the computational burden with the discrete Poisson model depends on the choice of temporal trend adjustment but in my experience has not resulted in prohibitively increased computational burden. The space-time permutation model does not suffer from increased computational burden with the addition of seasonal adjustments because adjustment for purely temporal trends including seasonality is automatic/implicit in the probability model.**

R4: We appreciate that in the reviewer’s experience, as well as in most of the authors’ experience, SaTScan software has no prohibitively high computational burden and can be effectively used in practise. We point out in our revised manuscript: “*SaTScan is a mature software widely used in disease surveillance*” (line 357).

We repeat here that SaTScan is a full software suite based on a statistical methodology, the scan statistics (while here we are presenting a backend algorithm), which has been developed over many years and often is a good choice, even considered a lower computational efficiency.

However, there are many situations where decreased computation burden may be extremely useful. As an example, this allowed us to sequentially run analyses to see how warning scores are updated prospectively as new cases are added (in the simulation experiment of Fig 6 we did that for 100 time points). Also this allows us to run analyses for different emm types; out cohort comprises 465 different emm types and subtypes, therefore even a moderate improvement in computational burden scales up significantly -- say, if one single analysis takes 1 hour less (which is not a decisive factor if one runs a single analysis overnight), running it 465 times might save up to 465 hours with respect to the slower method.

**C5: #11, re: “methods based on the scan statistics outputs an outbreak area of a determined shape such as a cylinder.”**

**- Effective SaTScan v.10.0 (see SaTScan version history under technical documentation), users can detect clusters along a network (see “Network file” in user guide), not only determined shapes such as circles or ellipses.**

R5: We thank the referee for pointing out this feature of SaTScan. We appreciate that SaTScan can also be used to look for clusters along network topology, such as a road network or a water distribution network. Since we focussed on cases occurring in time and geography, it is natural to compare our algorithm to classic spatio-temporal scan statistics.

The sentence cited above is part of our previous response letter, where we explain that our algorithm assesses single cases individually rather than outbreak regions***;*** this holds regardless of the underlying topology.

That sentence is not part of the manuscript, and the manuscript does not appear to make wrong statements in this regards. To improve clarity, in this minor revision, we included a sentence in the discussion (line 530): “*It will be interesting to explore and test variants of our methodology, similarly to how several variants of the scan statistics have been proposed (and implemented in statistical software) since the scan statistics was first introduced in 1965 [Naus1965a, Naus1965b]****”.***

**C6: Minor notes:**

**Intro: suggest clarifying common modes of transmission for iGAS outbreaks.**

R6: We included the following sentence in the Introduction: “*Transmission of GAS is generally person-to-person, involving large respiratory droplets and secretions, and direct or indirect contact with carriers through non-intact skin or mucous membranes.*”

**C7: Lines 83-84: suggest avoiding the term “epidemiologically linked” in this sentence as seemingly refers to only spatiotemporal proximity. I agree with the different use of this term in line 524.**

R7: We removed the word “epidemiologically” in that sentence.

**C8: Line 91: suggest clarifying what is meant by “appropriately” as I cannot infer what an inappropriate random collection of cylinders might look like.**

R8: To avoid confusion, we edited the sentence: “*To this end, we consider a neighbourhood of the point location and time of the recorded case and ~~appropriately~~ draw a random collection of spatio-temporal cylinders to cover it*. Line 91 (line 93 in the revised manuscript) is part of the introduction and the way cylinders are randomly sampled is detailed in the Methods section.

**C9: Line 257: How common is it for a single patient to be infected with multiple iGAS emm types?**

R9: It is extremely uncommon for invasive GAS infections. While infection with different organisms is possible, in our databases, which contains 10,000 recorded iGAS cases, there are no occurrences of patients infected with multiple iGAS emm type. We edited the manuscript accordingly after line 257.

**C10: Line 312, re: endemic cases “should not raise any public health concerns:” suggest rewording because high levels of preventable, endemic disease should be concerning to public health authorities, even in the absence of outbreaks.**

R10: We edited the sentence to “*should not raise ~~any~~* ***new*** *public health concerns***”**.

**C11: Fig. 1: Is It intentional that all circles have the same radius? Line 196 refers to "variable radia" so I would have expected the illustration to have different radii (and isn't the plural of radius radii, not radia?).**

R11: Yes, it is intentional that all the circles of Fig 1 have the same radius, since they represent sets of equal area and are depicted on a two-dimensional plane.

Analogously, we require that cylinders’ **volumes** in **space-time** are the same, with cylinders of constant volume obtained with varying cylinders’ heights and radii.

To clarify this, we edited caption of Fig 1: “*Representation of random neighbourhood covering* ***on a two-dimensional space***”, and text in line 195 of the revised manuscript: “***In space-time,*** *we opt to select cylinders of the same volume ∫A d ξ*”.

We also edited radia to radii all across the manuscript and we thank the reviewer for spotting the mistake.

**C12: Typos:**

**Line 25: “odd” should be “odds”**

**Line 291: “observation” should be “observations”**

**Line 815: “initial” should be “initially”**

**Line 816: “sample” should be “samples”**

R12: We thank the reviewer for spotting the typos. We edited the manuscript accordingly.

**Reviewer #2:**

**C1: I thank the authors for their thorough revision. In particular, being able to run the code for the simulation experiment is very valuable. The methods are also much clearer.**

**A couple details:**

R1: We thank the reviewer for their positive comments and suggestions.

**C2: -concerning your answer to my point for Figure 12, I agree that the highlight of the portion of A to which B corresponds is helpful, but I do not see a change in the x axes of panel B and D. I think shifting the plot of D to the right by one week, so that same dates are vertically aligned (in B and D) would make this plot more intuitive to read.**

R2: We thank the reviewer for their positive comments and for spotting our oversight in Fig 12. We edited it accordingly.

**C3: -I appreciate the addition of Figure S4, but don't forget to refer to it in the main text, linking it to Figure 6.**

R3: In the updated version of the manuscript, we reference Fig S4 in the caption of Fig 6.

“*Simulated outbreak points are close in both time and space, but it is hard to naively detect the anomaly (see also Figs S3 and S4)*”.
